# Effects of Dietary Alpha-Ketoglutarate Supplementation on Diarrhea Incidence and Nutrient Digestibility in Weaned Piglets Fed Low-Protein Diets

**DOI:** 10.3390/vetsci12121163

**Published:** 2025-12-06

**Authors:** Weiyan Sun, Ruyi Han, Kaikun Huang, Wenning Chen, Zhiwen Bian, Luca Marchetti, Hongbo Xi, Valentino Bontempo, Xianren Jiang

**Affiliations:** 1 Key Laboratory of Feed Biotechnology of Ministry of Agriculture and Rural Affairs, Institute of Feed Research, Chinese Academy of Agricultural Sciences, Beijing 100081, China; swy001103@163.com (W.S.); hhhry5201314@163.com (R.H.); bianzhiwen2024@163.com (Z.B.); 2 Shenzhen Xintianhe Biotechnology Co., Ltd., Shenzhen 518000, China; huangkaikun@kexing.com (K.H.); xihongbo@kexing.com (H.X.); 3 Department of Veterinary Medicine and Animal Science (DIVAS), University of Milan, 26900 Lodi, Italy; wenning.chen@unimi.it (W.C.); luca.marchetti1@unimi.it (L.M.); valentino.bontempo@unimi.it (V.B.)

**Keywords:** feed additive, growth, short-chain keto acid, weaning piglets

## Abstract

Alpha-ketoglutarate (AKG) supplementation has been explored as a nutritional strategy to mitigate the negative effects of protein restriction. This study aims to evaluate the effects of supplementing a low-protein diet with AKG on growth performance, diarrhea incidence, and fecal nutrient digestibility in weaned piglets. A total of 60 weaned piglets (initial body weight: 7.53 kg; age: 28 days old) were randomly distributed into three dietary treatments with five replicates per group, for a 42-day experimental period. The treatments consisted of a control diet (CT), a low-protein diet (LP), and a low-protein diet supplemented with 1000 g/t AKG (AKG). Fecal samples were collected on days 40–42 to determine the apparent digestibility of nutrients. The results showed that the feed conversion rate (FCR) in LP-group piglets were significantly higher than that of the CT group (*p* = 0.028), while the crude protein (CP) digestibility was significantly lower in the LP group compared with the CT group (*p* = 0.038). However, supplementing the low-protein diet with 1000 g/t AKG effectively mitigated these negative effects. Pigs in the AKG group had reduced incidence of diarrhea between days 1 and 14 compared to CT group (*p* = 0.041). These findings suggest that incorporating 1000 g/t AKG into low-protein diets may reduce post-weaning diarrhea and improve growth performance of piglets.

## Simple Summary

Low-protein diets are designed to meet animals’ amino acid requirements while reducing dietary crude protein levels, offering several benefits such as decreased feed costs, lower nitrogen emissions, and reduced environmental pollution. However, such diets may compromise animal growth performance in some cases. Alpha-ketoglutarate (AKG), a key intermediate of the tricarboxylic acid (TCA) cycle, plays an important role in energy metabolism and amino acid synthesis. In this study, dietary supplementation with 1000 g/t AKG has been shown to mitigate these negative effects by reducing the incidence of diarrhea in piglets fed low-protein diets.

## 1. Introduction

Nitrogen emissions from livestock farms, primarily originating from animal manure and urine [1], are a major contributor of environmental pollution. Fecal nitrogen comes from undigested dietary protein, which is vulnerable to microbial degradation producing poisonous and harmful gases such as ammonia, hydrogen sulfide, and sulfur dioxide, which contribute to respiratory diseases in pigs [2]. Urinary nitrogen is the main form of urea, the urea enzyme decomposition of ammonium, and is subject to carbonate, eventually polluting the environment [3].

It has been demonstrated that reducing dietary protein content while balancing amino acids profiles could effectively decrease the total nitrogen and urinary nitrogen excretion of growing-finishing pigs, thereby reducing ammonia emissions in pig housing [4,5]. However, some research has also pointed out that reducing the protein level in the daily diet by more than 3% may restrict the growth performance of pigs [6,7]. High-protein diets are also known to promote the proliferation of enteropathogenic Escherichia coli and increase the incidence of post-weaning diarrhea in piglets [8]. Therefore, formulating low-protein (LP) diets with balanced amino acids has attracted increasing attention as a nutritional strategy to improve gut health and reduce environmental nitrogen emissions.

Alpha-ketoglutarate (AKG) is a non-toxic, short-chain carboxylic acid that acts as a central component of the tricarboxylic acid (TCA) cycle. Its involvement spans energy metabolism, nitrogen utilization, and the regulation of intestinal health [9,10]. Studies have shown that dietary addition of AKG can mitigate the negative effects associated with reduced protein levels [11]. Our previous research confirmed that supplementing piglet diets with 1000 g/t of AKG reduced diarrhea incidence and improved health status in weaned piglets [12].

However, the effects of AKG supplementation in low-protein diets on growth performance, diarrhea incidence and fecal apparent nutrient digestibility in weaned piglets remain unclear. This study hypothesizes that adding AKG to the diet may alleviate the adverse effects of low-protein diet, providing a scientific data basis for its application in piglets’ diets.

## 2. Materials and Methods

### 2.1. Animal Ethics Approval

Approval for all animal experiments was obtained from the Animal Care and Use Committee of the Institute of Feed Research, Chinese Academy of Agricultural Sciences (IFR-CAAS20240302). The trial took place at the Langfang Experimental Farm located in Hebei Province, China.

### 2.2. Experimental Design

Sixty weaned barrows (Duroc × Landrace × Yorkshire) with mean initial body weight of 7.53 ± 0.57 kg and an average age of 28 ± 2 days were randomly assigned to three dietary treatments in a 42-day trial, with five replicate pens per treatment and four piglets per pen. Phase 1 and Phase 2 diets were fed from days 0–14 and days 14–42, respectively. All piglets were obtained from a commercial farm in Langfang, Hebei Province, and housed in a nursery facility.

The control group (CT) received a corn–soybean meal–based diet, the low-protein group (LP) had its protein level reduced by 2.5%, and the AKG group was provided with the same low-protein diet supplemented with 1000 g/t of α-ketoglutaric acid (purity ≥ 99%, supplied by Sinovac Biotech Ltd., Shenzhen, China). Dietary amino acid profiles were balanced by adding crystalline amino acids (valine, lysine, methionine, threonine, tryptophan and isoleucine) to fulfill the nutritional requirements of piglets.

The ambient temperature was initially maintained at 28 °C and gradually reduced by 1 °C each week until 26 °C, after which it was kept at 26 °C for the remainder of the trial, relative humidity was kept within the range of 55–65%. Feed and water were available to the piglets *ad libitum*. Vaccinations were administered according to routine farm procedures, and the pens were cleaned regularly throughout the trial. Experimental diets were formulated to meet or exceed the NRC (2012) recommendations for growing pigs (5–11 kg body weight) [13]. Minor adjustments were made based on ingredient analyses to accommodate the evaluation of AKG supplementation, while maintaining consistent basal nutrient concentrations across all groups. And the ingredient composition and calculated nutrient levels are presented in Table 1.

### 2.3. Growth Performance and Diarrhea Incidence

The body weight (BW) of each piglet was individually measured on days 0, 14, 28, and 42 of the experiment. Feed was offered once daily at 8:00 a.m., and the residual feed in the troughs was weighed to determine daily feed intake. Average daily gain (ADG), average daily feed intake (ADFI), and feed conversion ratio (FCR) were calculated from the recorded data and used to characterize the growth performance of piglets. From day 1 to day 14, fecal scores was evaluated daily to monitor the occurrence of diarrhea. Each piglet was assessed individually at 9:00 a.m. using a five-point fecal scoring system:

1 = hard, granular feces;

2 = hard, well-formed feces;

3 = soft but shaped feces;

4 = soft, unformed feces;

5 = watery feces.

Scores of 4 or 5 were defined as diarrhea.

Diarrhea incidence (%) = Number of diarrheic piglets/(Total piglets × Experimental days) × 100.

### 2.4. Apparent Digestibility of Nutrients

Fecal samples were collected from all piglets at the same time on days 40, 41, and 42 of the trial. During each collection, fresh feces excreted by all piglets within a pen were gathered into a clean box and thoroughly mixed to form one composite sample per pen. Immediately after collection, the samples were stored at −20 °C to minimize nitrogen loss and microbial degradation. Samples were oven-dried at 65 °C for 72 h and subsequently analyzed for apparent nutrient digestibility, which was calculated using acid-insoluble ash (AIA) as an internal marker, and its concentration in both diets and feces was analyzed according to the described method [14].

The diets and fecal samples were analyzed for dry matter (DM; method 930.15), ether extract (EE; method 920.39A), and crude protein (CP; N × 6.25; method 990.03) following the procedures outlined in AOAC (2005) [15]. Gross energy (GE) was measured using a Parr 6400 calorimeter (Parr Instrument Company, Moline, IL, USA).

### 2.5. Statistical Analysis

Statistical analyses of growth performance and nutrient digestibility were performed using one-way ANOVA in SAS 9.4 (SAS Institute Inc., Cary, NC, USA). Results are expressed as means ± SEM. The chi-square test was applied to evaluate the incidence of diarrhea. Differences were considered statistically significant at *p* < 0.05.

## 3. Results

### 3.1. Growth Performance and Diarrhea Incidence

Table 2 shows the influence of AKG supplementation in low-protein diet on growth performance of weaned piglets. Compared with the CT group, no significant difference was observed in BW, ADG, and ADFI in piglets fed a low-protein diet (*p* > 0.05), but FCR was significantly higher in the LP group (*p* = 0.028), and the AKG group showed no significant difference with CT group.

**Figure 1:**
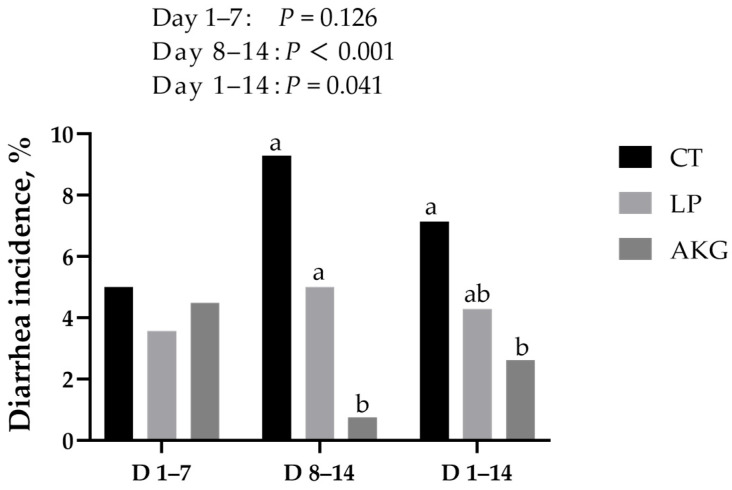
Supplementation of AKG in low-protein diet on diarrhea incidence of weaned piglets. ^a,b^ Values bearing different superscripts are considered significantly different (*p* < 0.05).

Figure 1 demonstrates the effect of AKG supplementation in low-protein diet on diarrhea incidence of weaned piglets. From day 1 to 14, the incidence of diarrhea was not significantly different between the CT group and the LP group, while the AKG group showed a significant decrease in diarrhea incidence compared with the control group (*p* = 0.041).

### 3.2. Nutrient Apparent Digestibility

Table 3 illustrates the influence of AKG supplementation in low-protein diet on nutrient apparent digestibility of weaned piglets. No significant difference was observed in apparent digestibility of DM, EE and GE between all the experimental groups. Compared with the CT group, the CP digestibility of piglets fed a low-protein diet was significantly reduced (*p* = 0.038), and the CP digestibility in the AKG group showed no significant difference with CT group.

## 4. Discussion

Post-weaning diarrhea is a major challenge affecting production efficiency in the swine farming industry. After weaning, piglets exhibit an immature gastrointestinal tract, characterized by insufficient secretion and low activity of digestive enzymes, which restricts the efficient digestion and absorption of nutrients, particularly dietary proteins. Plant-derived protein sources, especially soybean meal, although regarded as high-quality protein ingredients, contain various antinutritional factors and antigenic proteins that are poorly tolerated by the immature gut of weaning piglets [16]. Studies have shown that when dietary CP levels are excessively high (above 20%), the presence of large amounts of antigenic proteins in plant-based proteins may increase intestinal permeability and compromise the integrity of the intestinal barrier [17], thereby reducing nutrient absorption efficiency and increasing the risk of post-weaning diarrhea. These alterations can impair nutrient absorption and contribute to a higher incidence of post-weaning diarrhea. Numerous studies have demonstrated that low-protein diet can effectively reduce the occurrence of diarrhea after weaning [18,19,20]. Although the low-protein diet alone did not significantly reduce the incidence of diarrhea, which may be attributed to the overall low occurrence of diarrhea in this study, the addition of 1000 g/t AKG to the low protein diet significantly reduced the incidence of diarrhea in weaned piglets. This result is consistent with our previous study [12], and this appearance may be related to improved CP digestibility.

Although reducing the protein levels in the diet may reduce the incidence of diarrhea in piglets, low-protein diets may also potentially limit the growth performance of pigs. Studies have reported that lowering dietary protein decreases the ADG of pigs and significantly increases the FCR [21,22,23]. The results of the present study are consistent with these reports, as the FCR of piglets in the LP group was significantly higher than that in the CT group, while supplementation with 1000 g/t AKG partially restored FCR. These results may indicate that supplementation with 1000 g/t of AKG may alleviate the increase in FCR caused by a low-protein diet. However, recent research has also found that low-protein diets do not always impair the growth performance of pigs [24,25], which may be related to the age of weaning, the weight, feed composition, and growth environment. This inconsistency highlights the importance of considering both diet formulation and functional supplementation strategies to optimize growth performance under low-protein feeding. It should be noted that fecal samples were collected only during the late conservation period (days 40–42), which may not fully reflect the dynamic changes in nutrient digestibility during the early post-weaning phase. Future studies should include sampling at multiple stages to provide a more comprehensive assessment of digestive adaptation.

The digestion and absorption of protein are closely linked to the growth performance of piglets. In our study, the CP digestibility of piglets fed a low-protein diet was significantly lower than that of the CT group, while supplementation with 1000 g/t AKG improved digestibility. This result is supported by previous studies [26,27], which reported that even when low-protein diets were supplemented with amino acids, digestibility remained lower than in the control group. This reduction may be partially explained by differences in endogenous nitrogen losses. When dietary protein content is low, the proportion of endogenous nitrogen (derived from digestive enzymes, sloughed epithelial cells, and mucin proteins) increases relative to total fecal nitrogen, thereby reducing the apparent CP digestibility [28,29]. In addition, the dietary protein level can influence the secretion and activity of proteolytic enzymes in the gastrointestinal tract. Within a certain physiological range, the apparent CP digestibility tends to be positively correlated with dietary protein concentration [30]. As an intermediate in the Krebs cycle, mitochondrial AKG participates in oxidative metabolism, producing carbon dioxide (CO_2_) and nicotinamide adenine dinucleotide with hydrogen (NADH) for energy. AKG can be converted to glutamate by glutamate dehydrogenase (GDH), and then to glutamine by glutamine synthase (GS), supporting nitrogen metabolism and protein synthesis [31,32]. Moreover, the research also demonstrated that supplementing AKG in low-protein diets significantly increased the apparent nitrogen digestibility and net protein utilization in growing pigs [33]. Dietary supplementation with AKG can activate the mTOR signaling pathway, alleviate oxidative stress and damage in intestinal mucosal cells, and improve intestinal mucosal integrity and nutrient absorption in pigs challenged with endotoxins [9,34]. It can also mitigate intestinal inflammation, enhance epithelial repair under stress, and support gut health during the early post-weaning period [35]. Moreover, AKG supplementation modulates the gut microbiota by promoting the growth of beneficial bacteria, increasing concentrations of butyrate and valine, and reducing intestinal ammonia levels in growing pigs [36]. Based on the above results, supplementing 1000 g/t AKG in a low-protein diet may improve CP digestion, thereby reducing diarrhea incidence in weaned piglets. It should also be noted that most orally administered AKG is likely metabolized within intestinal tissues, implying that its major effects are localized in the gut [37]. Nevertheless, a portion may enter systemic circulation and contribute to metabolic regulation in peripheral tissues [38]. Future studies should therefore quantify plasma AKG, glutamate, and glutamine concentrations to distinguish local versus systemic effects and further clarify the mode of action of AKG in pigs.

However, it should be noted that the CP digestibility measured in this study represents only apparent total-tract digestibility and does not accurately reflect the true absorption of amino acids, which primarily occurs in the upper small intestine [39]. Moreover, the reduction in dietary CP levels may have increased the relative proportion of endogenous nitrogen losses, thereby decreasing the apparent CP digestibility of the low-protein diets [40]. Future studies should include ileal amino acid digestibility measurements or estimates of endogenous nitrogen losses to more accurately assess true protein utilization in piglets.

## 5. Conclusions

The effects of low-protein diets on growth and nutrient digestibility in piglets have been reported inconsistently across studies. Supplementing a low-protein diet with 1000 g/t AKG partially mitigated the negative effects associated with reduced dietary protein levels. AKG supplementation significantly decreased diarrhea incidence during the early post-weaning period, although no significant improvement in growth performance was observed. These findings suggest that dietary AKG may help alleviate weaning-associated intestinal challenges and support nutrient utilization in piglets fed low-protein diets.

## Figures and Tables

**Table 1:** Ingredient composition of the diets (%, as-fed basis).

	Phase 1 (Day 0–14)			Phase 2 (Day 14–42)		
	CT	LP	AKG	CT	LP	AKG
Corn	46.46	52.33	52.23	60.30	65.23	65.13
Soybean meal, 46%	13.20	8.50	8.50	17.30	11.40	11.40
Expanded soybean	14.50	13.50	13.50	6.50	6.00	6.00
Fish meal	6.00	4.50	4.50	4.00	3.00	3.00
Whey powder	15.00	15.50	15.50	5.00	6.70	6.70
Soybean oil	1.10	1.00	1.00	1.20	1.00	1.00
Wheat bran	0.11	0.11	0.11	2.14	2.14	2.14
Calcium dihydrogen phosphate	0.35	0.55	0.55	0.60	0.70	0.70
Limestone	0.77	0.85	0.85	1.00	1.05	1.05
Salt	0.40	0.40	0.40	0.40	0.40	0.40
Choline chloride, 60%	0.05	0.05	0.05	0.05	0.05	0.05
L-Lysine HCL, 65%	0.90	1.20	1.20	0.65	1.05	1.05
DL-Met	0.06	0.11	0.11	0.05	0.10	0.10
Threonine	0.24	0.30	0.30	0.14	0.25	0.25
Tryptophan	0.02	0.06	0.06	0.01	0.06	0.06
Valine	0.00	0.10	0.10	0.02	0.15	0.15
Isoleucine	0.00	0.10	0.10	0.00	0.08	0.08
Phytase (10,000) ^1^	0.02	0.02	0.02	0.02	0.02	0.02
Acidifier ^2^	0.20	0.20	0.20	0.20	0.20	0.20
Sodium butyrate	0.15	0.15	0.15	0.15	0.15	0.15
Alpha-ketoglutarate (AKG)	0.00	0.00	0.10	0.00	0.00	0.10
Zinc oxide	0.02	0.02	0.02	0.00	0.00	0.00
Premix ^3^	0.45	0.45	0.45	0.27	0.27	0.27
Sum	100	100	100	100	100	100
Nutrition composition						
Analyzed value						
Crude protein, CP, %	19.55	16.94	16.99	17.97	15.59	15.62
Calcium, Ca, %	0.82	0.81	0.81	0.73	0.72	0.72
Phosphorus, P, %	0.53	0.53	0.54	0.47	0.49	0.50
Ether extract, EE, %	5.34	5.27	5.28	5.92	5.62	5.64
Calculated value ^4^						
ME, MJ/kg	14.00	14.00	14.00	13.81	13.81	13.81
Lys, %	1.25	1.25	1.25	1.22	1.22	1.22
Met, %	0.36	0.36	0.36	0.35	0.35	0.35
Thr, %	0.74	0.74	0.74	0.72	0.72	0.72
Trp, %	0.21	0.21	0.21	0.21	0.21	0.21
Val, %	0.78	0.78	0.78	0.77	0.77	0.77
Ile, %	0.64	0.64	0.64	0.63	0.63	0.63

^1^ Phytase (10,000) was provided by Inner Mongolia Yiduoli Biotechnology Co., Ltd. (Hohhot, China), with a minimum activity of 10,000 U/g and moisture ≤ 10%. ^2^ Acidifier was provided by Anhui Zhengzheng Feed Technology Co., Ltd. (Hefei, China) China. Its raw material composition included citric acid, fumaric acid, formic acid, and benzoic acid. Guaranteed analysis: benzoic acid ≥ 20.1%, citric acid ≥ 16.2%, formic acid ≥ 16.0%, fumaric acid ≥ 4.2%; total acidity (as lactic acid) ≥ 70%, moisture ≤ 12%. ^3^ Premix supplied per kg of diet: vitamin A, 35.2 mg; vitamin D_3_, 7.68 mg; vitamin E, 128 mg; vitamin K_3_, 8.16 mg; vitamin B_1_, 4 mg; vitamin B_2_, 12 mg; vitamin B_6_, 8.32 mg; vitamin B_12_, 4.8 mg; Niacin, 38.4 mg; Calcium pantothenate, 25 mg; Folic acid, 1.68 mg; Biotin, 0.16 mg; Zn (ZnSO_4_·H_2_O), 110 mg; Copper (CuSO_4_·5H_2_O), 125 mg; Iron (FeSO_4_·H_2_O), 171 mg; Cobalt (CoCl_2_), 0.19 mg; Manganese (MnSO_4_·H_2_O), 42.31 mg; Iodine (Ca(IO_3_)_2_), 0.54 mg; Selenium (Na_2_SeO_3_), 0.19 mg. ^4^ Calculated amino acid values were derived from feed ingredient composition and analyzed total amino acid content. Standardized ileal digestible (SID) values were not measured.

**Table 2:** Supplementation of AKG in low-protein diet on growth performance of weaned piglets.

	CT	LP	AKG	SEM	*p*-Value
BW, kg ^1^					
Day 0	7.54	7.52	7.52	0.57	0.551
Day 14	12.27	11.98	12.17	0.82	0.774
Day 28	19.55	18.43	18.88	1.22	0.290
Day 42	28.31	25.87	26.93	1.60	0.166
ADG, g					
Day 0–14	338	318	333	23	0.799
Day 14–28	520	461	479	34	0.304
Day 28–42	625	532	575	36	0.218
Day 0–42	494	437	462	27	0.173
ADFI, g					
Day 0–14	450	469	486	33	0.540
Day 14–28	799	768	767	66	0.853
Day 28–42	1107	943	983	55	0.137
Day 0–42	785	726	745	46	0.413
FCR					
Day 0–14	1.330 ^b^	1.483 ^a^	1.463 ^a,b^	0.043	0.028
Day 14–28	1.542	1.686	1.590	0.085	0.661
Day 28–42	1.768	1.780	1.718	0.042	0.573
Day 0–42	1.588	1.661	1.611	0.028	0.319

^1^ BW, Body weight; ADG, Average Daily Gain; ADFI, Average Daily Feed Intake; FCR, Feed Conversion Ratio. Animals were weighed using a scale with precision up to 0.01 kg; values are reported to two decimal places. ^a,b^ Values in the same row carrying different superscripts indicate a statistically significant difference (*p* ≤ 0.05).

**Table 3:** Supplementation of AKG in low-protein diet on nutrient apparent digestibility (%) of weaned piglets.

	CT	LP	AKG	SEM	*p*-Value
Dry matter	78.66	77.72	78.48	0.66	0.581
Crude protein	75.21 ^a^	70.89 ^b^	72.79 ^ab^	1.00	0.038
Ether extract	69.05	67.76	69.60	2.32	0.850
Gross energy	80.53	79.53	80.26	0.75	0.640

^a,b^ Values in the same row carrying different superscripts indicate a statistically significant difference (*p* ≤ 0.05).

## Data Availability

The original contributions presented in this study are included in the article. Further inquiries can be directed to the corresponding author(s).

## Abbreviations

The following abbreviations are used in this manuscript:

ADFI	Average Daily Feed Intake
ADG	Average Daily Gain
AIA	Acid insoluble ash
AKG	Alpha-ketoglutaric acid
CO_2_	Carbon dioxide
CP	Crude protein
DM	Dry matter
EE	Ether extract
FCR	Feed Conversion Ratio
GDH	Glutamate dehydrogenase
GE	Gross energy
GS	Glutamine synthase
NADH	Nicotinamide adenine dinucleotide with hydrogen
